# Increased newborn NICU admission for evaluation of hypoxic-ischemic encephalopathy during COVID-19 pandemic in a public hospital

**DOI:** 10.3389/fped.2023.1206137

**Published:** 2023-06-29

**Authors:** Dongli Song, Sudha Rani Narasimhan, Angela Huang, Priya Jegatheesan

**Affiliations:** ^1^ Department of Pediatrics, Division of Neonatology, Santa Clara Valley Medical Center, San Jose, CA, United States; ^2^ Department of Pediatrics, Stanford University School of Medicine, Stanford, CA, United States

**Keywords:** COVID-19 pandemic, HIE (hypoxic ischaemic encephalopathy), therapeutic hypothermia, metabolic acidosis, maternal hypertension

## Abstract

**Background:**

Prenatal and perinatal care of pregnant mothers has been adversely affected during the COVID-19 pandemic. Hypoxic-ischemic encephalopathy (HIE) is a leading cause of neonatal death and long-term neurological disabilities. Therapeutic hypothermia is effective for neonatal HIE. This study evaluated the effect of the pandemic on neonatal HIE.

**Methods:**

This retrospective single-center study compared neonatal HIE evaluation and hypothermia treatment between pre-COVID-19 pandemic (1 January 2018–31 December 2019) and COVID-19 pandemic (1 January 2020–31 December 2021) periods. Infants with abnormal neurological examination and or significant metabolic acidosis were admitted to NICU for evaluation of HIE and therapeutic hypothermia. Demographics, NICU admission and interventions, and neonatal outcomes were compared between infants born during the two periods using *χ*^2^, *t*-test, and Wilcoxon rank-sum test as appropriate. Statistical Process Control charts show the yearly proportion of infants evaluated for HIE and those treated with therapeutic hypothermia.

**Results:**

From the pre-pandemic to the pandemic period, the proportion of infants that met HIE screening criteria increased from 13% to 16% (*p* < 0.0001), the proportion of infants admitted to NICU for HIE evaluation increased from 1% to 1.4% (*p* = 0.02), and the maternal hypertension rates of the admitted infants increased from 30% to 55% (*p* = 0.006). There was no difference in the proportions of the infants diagnosed with HIE (0.7% vs. 0.9%, *p* = 0.3) or treated with therapeutic hypothermia (0.2% vs. 0.3%, *p* = 0.3) between the two periods. There were no differences in the HIE severity and outcomes of the infants treated with therapeutic hypothermia between the two periods.

**Conclusion:**

During the COVID-19 pandemic, we observed a significant increase in NICU admission for HIE evaluation. While we did not find significant increases in neonatal HIE and the need for therapeutic hypothermia, larger studies are needed for a comprehensive assessment of the impact of the COVID-19 pandemic on neonatal HIE.

## Introduction

Neonatal encephalopathy is a leading cause of infant mortality and long-term neurodevelopmental abnormalities (1–4). It is a clinically defined syndrome of newborns manifested by an abnormal level of consciousness or seizures, often accompanied by difficulty with initiating and maintaining respiration and depression of tone and reflexes (5). Hypoxic-ischemic encephalopathy (HIE) is the subset of neonatal encephalopathy with evidence of a recent hypoxic–ischemic cause of the encephalopathy. Therapeutic hypothermia (TH), initiated within the first 6 h of life, is the only proven effective neuroprotective therapy for moderate and severe neonatal HIE (6–10). Timely evaluation of newborns at risk of HIE and early initiation of TH is critical for better outcomes (11, 12).

The COVID-19 pandemic has had a profound impact on prenatal and perinatal care. While COVID-19 infection during pregnancy directly increases maternal and neonatal morbidity and mortality (13–16), the indirect factors associated with the pandemic also adversely affect maternal health, pregnancy, and neonatal outcomes (17). During the COVID-19 pandemic, many aspects of the healthcare system were disrupted, and pregnant women experienced fear, mental stress, and a worsening in socioeconomic disparities (17, 18). Many studies have shown increased pregnancy complications, including maternal diabetes, hypertension, and obesity during the pandemic (19–26). These complications are known risk factors for perinatal sentinel events and fetal and neonatal hypoxic-ischemic injury (27–30).

To date, there is limited information on the effect of the COVID-19 pandemic on neonatal HIE. Our institution established standardized protocols for HIE screening, evaluation, and TH in 2008. This study compared our HIE data between the pre-COVID-19 and COVID-19 periods to assess the effect of the pandemic on the incidence and outcomes of neonatal HIE.

## Methods

### Study design and subjects

This is a single-center, retrospective, observational study conducted in a public safety-net hospital. The study includes two time periods: the pre-COVID-19 period (January 2018–December 2019) and the COVID-19 period (January 2020–December 2021). We included infants born at ≥35 weeks gestation during the study periods. Infants with no intent to resuscitate or those who died in the delivery room were excluded. The study was approved by the institutional review board.

Standardized protocols for HIE screening, evaluation and therapeutic hypothermia in infant ≥35weeks GA

**Figure 1:**
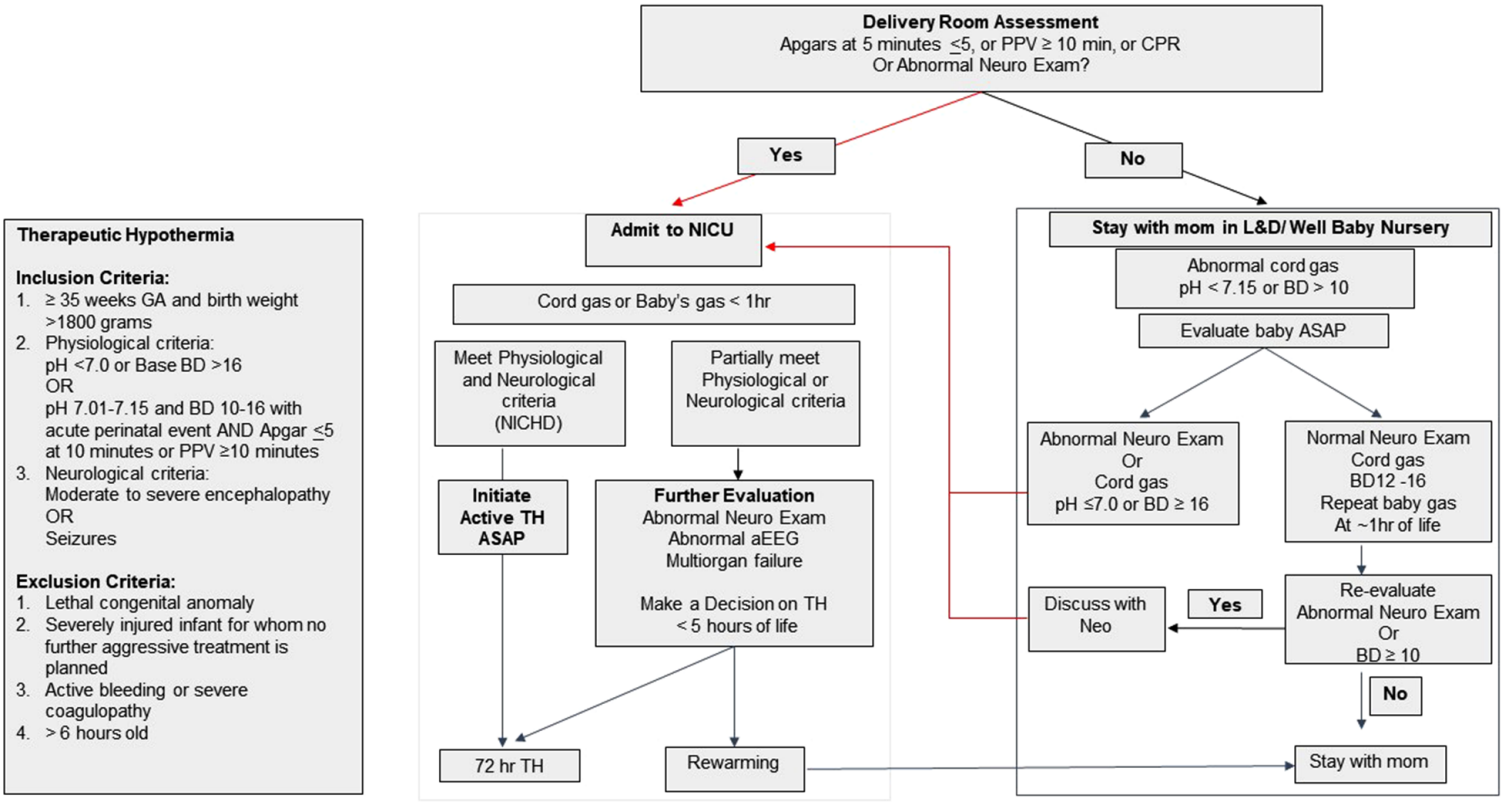
Hypoxic ischemic encephalopathy (HIE) screening and evaluation flow chart and criteria for initiating therapeutic hypothermia (TH).

Our standardized neonatal HIE evaluation and total body TH protocols were established in 2008 based on the published multicenter randomized controlled trials (6, 7), which have not been changed since then. Cord blood gas (CBG) with pH <7.15 and/or base deficit (BD) >10 mmol/L are reported to NICU immediately. The HIE screening and evaluation process is shown in Figure 1. HIE screening criteria includes a history of perinatal sentinel hypoxic or ischemic events, DR resuscitation with chest compressions or positive pressure ventilation ≥10 min, 5 min Apgar scores ≤5, abnormal neurological examination at birth, and/or CBG with pH <7.15 and/or BD > 10 mmol/L. Infants who meet the screening criteria are examined by pediatric providers. Infants with abnormal neurological examination and or CBG pH <7.0 and/or BD > 16 mmol/L are admitted to NICU for HIE evaluation and treatment. Infants who have normal neurological examination at birth, but with persistent metabolic acidosis >10 mmol/L or if neurological examination becomes abnormal during re-evaluation at one hour of life are admitted to NICU. Infants who meet criteria for TH (Figure 1) are cooled as soon as possible. Infants with an abnormal neurological examination but do not meet the TH criteria on initial assessment continue to be monitored and evaluated for up to five hours of life. Ongoing evaluation includes follow-up infant blood gas within the first hour of life, serial neurological examinations, bedside two-channel (C3-P3, C4-P4) aEEG monitoring (Natus Medical, Middleton, WI, USA), and laboratory tests for assessing multiorgan injuries (29, 31). Hypothermia is initiated in infants with persistent or worsening neurological examination, abnormal aEEG (with raw EEG tracing) and/or evidence of multiorgan injuries. HIE evaluation ends if the neurological examination is normalized during the first hours of life.

Total body TH is performed according to the published method (6). During TH, infants' neurological status is assessed by daily neurological examination, continuous bedside aEEG/EEG, and cerebral O2 saturation monitoring. These infants are evaluated and followed by pediatric neurology service, including full channel video EEG evaluation. Brain MRI is performed after TH is completed and when the infant is medically stable to assess brain injury, generally on day of life 4–7. Brain MRI is reviewed by a pediatric radiologist or a neuroradiologist. The severity of brain injury is scored using the scoring system published by Barkovich et al. (32).

### Data collection

Maternal and infant demographics, maternal hypertension (chronic hypertension in pregnancy, and gestational hypertension and preeclampsia) (33, 34), diabetes (pre-gestational and gestational) (35, 36), infection, perinatal events, DR interventions, HIE screening, NICU admission for HIE evaluation, TH, and length of NICU stay were obtained from NICU database and electronic medical records.

Demographics, DR measures, NICU interventions, and neonatal outcomes were compared between infants admitted to NICU for HIE evaluation during pre-pandemic and pandemic periods using *χ*^2^, Fisher's exact, *t*-test, Wilcoxon (Mann–Whitney) rank-sum test as appropriate. STATA 14.0 (Statacorp, TX, USA) was used for statistical analysis. A *p*-value <0.05 was considered significant. We used Statistical Process Control (QI macros 2019 Excel add-on software, KnowWare International, Denver, CO, USA) to show the yearly proportion of infants evaluated for HIE in NICU and infants treated with TH as p charts.

## Results

During the four-year study period, there were 10,956 infants born at GA of ≥35 weeks (pre-COVID-19, *n* = 5,638, COVID-19, *n* = 5,318) (Table 1) at our institution. Of these deliveries, 3,910 CBG were done, and there was no difference in the percentages of CBG between the two periods (35% vs. 36%, *p* = 0.7). From the pre-COVID-19 period to the COVID-19 period, the proportion of infants that met HIE screening criteria increased from 13% to 16% (*p* < 0.0001), and the proportion of infants admitted to NICU for HIE evaluation increased from 1% to 1.4% (*p* = 0.02). There was no difference in the proportions of infants diagnosed with HIE (0.7% vs. 0.9%, *p* = 0.3) or treated with TH (0.2% vs. 0.3%, *p* = 0.3) between the two periods.

**Table 1:** Hypoxic ischemic encephalopathy screening and evaluation .

	Pre-COVID-19 (2018–2019)	COVID-19 (2020–2021)	*p*-value
Birth ≥35 weeks GA, *n*	N = 5,638	N = 5,318	
Cord blood gas obtained, *n* (%)	2,001 (35)	1,909 (36)	0.7
Cord/infant blood gas met screening criteria (pH ≥7.15 or BD ≥ 10), *n* (%)	733 (13)	851 (16)	**<0.0001**
Infants admitted to NICU for HIE evaluation, *n* (%)	54 (1)	77 (1.4)	**0.02**
Infants diagnosed with HIE, *n* (%)	38 (0.7)	45 (0.9)	0.3
Infants treated with therapeutic hypothermia, *n* (%)	12 (0.2)	18 (0.3)	0.3

GA, gestational age; HIE, hypoxic ischemic encephalopathy.

The bold values are statistically significant (*p* < 0.05).

**Figure 2:**
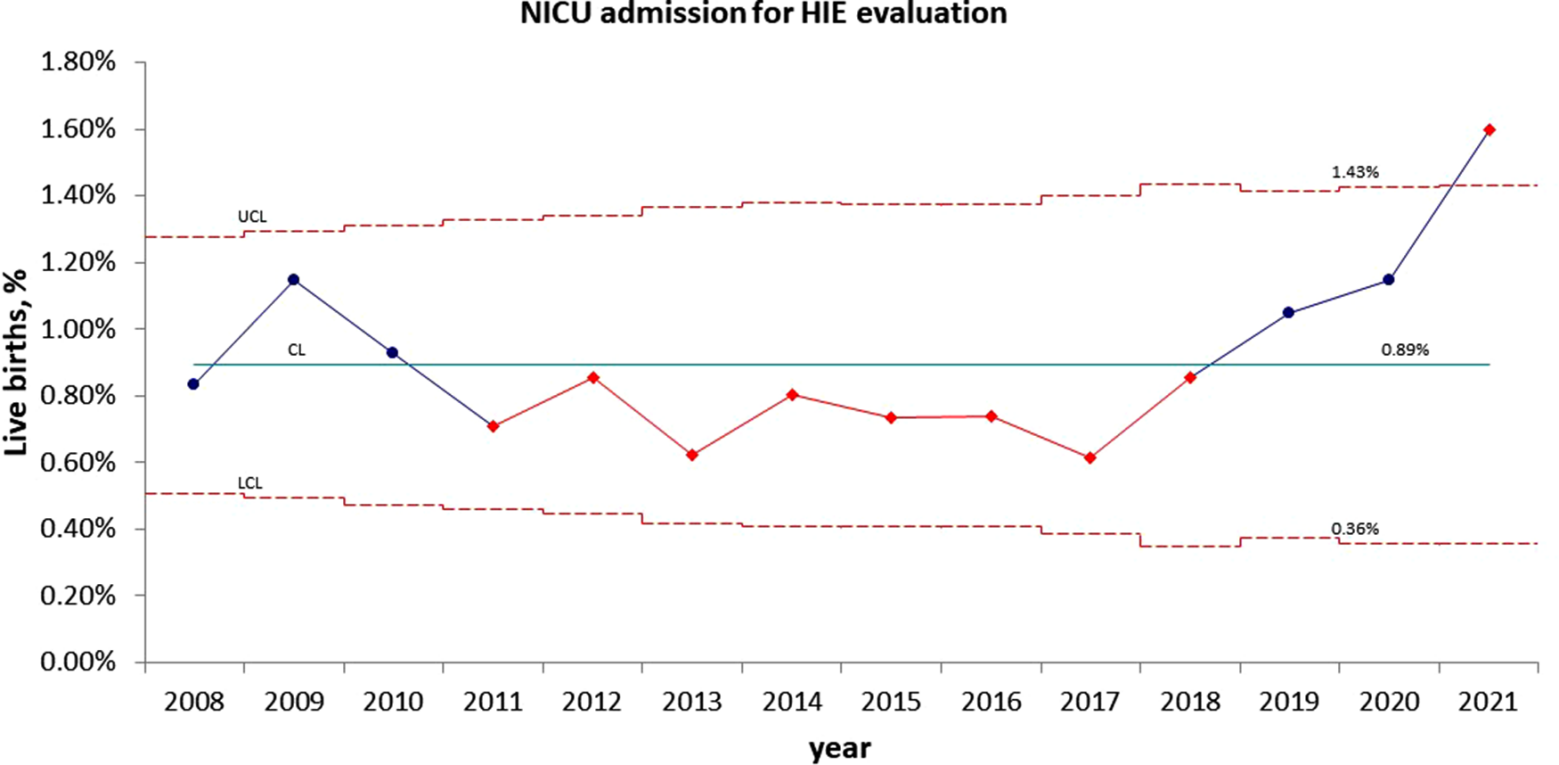
Statistical process control (p) chart shows the percent of infants admitted to NICU for evaluation of hypoxic ischemic encephalopathy (HIE) each year. The central line (solid) represents the mean and upper and lower control limit lines (dashed) represent 3 standard deviations from the mean.

**Figure 3:**
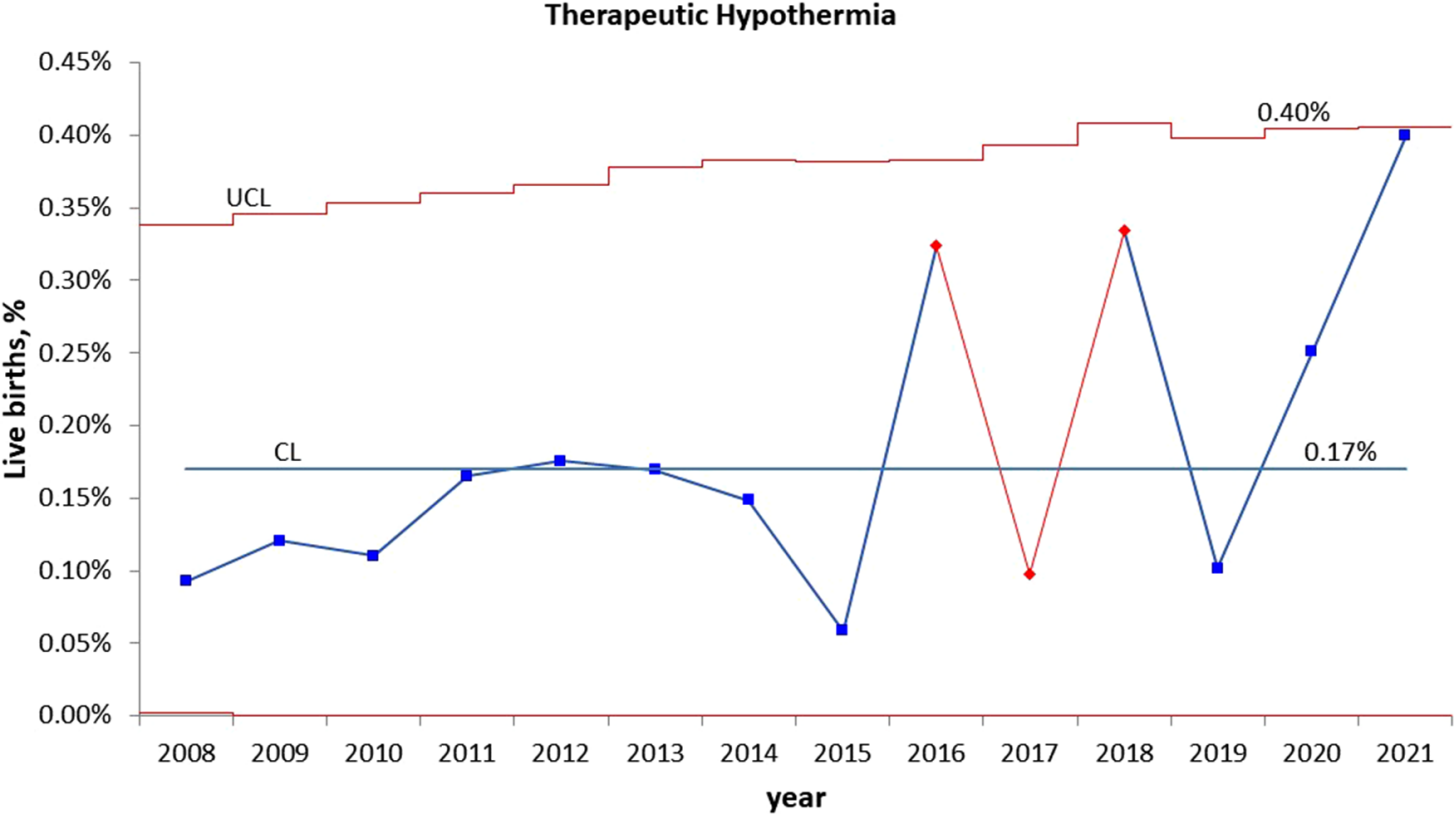
Statistical process control (p) chart shows the percent of infants treated with therapeutic hypothermia for hypoxic ischemic encephalopathy (HIE) each year. The central line (solid) represents the mean and upper and lower control limit lines (dashed) represent 3 standard deviations from the mean.

Statistical process control charts show a significant increase in the percentage of infants admitted to NICU for HIE evaluation in year 2021 (Figure 2) but no significant increase in the percentage of infants who received TH during pandemic years (Figure 3).

The maternal and infant demographics and clinical characteristics of the 131 cases that required NICU admission for HIE evaluation are shown in Table 2. During the COVID-19 period, there was an increase in maternal hypertension from 30% to 55% (*p* = 0.006). There were no differences in other pregnancy morbidities or delivery complications. Three mothers had asymptomatic or mild COVID-19 infection during pregnancy, and their newborns tested negative for SARS-Co-2 virus. During the COVID-19 period, there was a reduction in BW (−297 grams, *p* = 0.005) and a 7% increase in small for GA (SGA), although not statistically significant (*p* = 0.09). During the pre-COVID-19 period, six infants (11%) received chest compressions (CPR) compared to one infant (1%) in the COVID-19 period. Three of the six infants who received CPR had brief CPR for 30–60 s and were not intubated. One and five minute Apgar scores in the COVID-19 period were lower than the COVID-19 period. However, Apgar scores ≤5 were not different between the two periods. There was no difference in infants who had pH <7.0 in the CBG or first infant blood gas between the two periods, but there was an increase in infants with severe metabolic acidosis in the COVID-19 period (BD > 12 mEg/L: 22%–55%, *p* < 0.0001). There was no difference in abnormal neurological examination at one hour of life and multiorgan failure between the two periods. There were no differences in the HIE severity and short-term outcomes of the infants treated with TH between the two periods (Table 3).

**Table 2:** Infants admitted to NICU for hypoxic ischemic encephalopathy evaluation.

	Pre-COVID-19 (2018–2019) *N* = 54	COVID-19 (2020–2021) *N* = 77	*p*-value
**Maternal Demographics**			
Maternal age, Mean (SD)	31.1 (6.8)	31.4 (7.3)	0.8
Gravida, Median (IQR)	2 (1, 4)	3 (1.5, 4)	0.6
Para, IQR)	2 (1, 3)	2 (1, 3)	0.9
Multiples, *n* (%)	0	4 (5)	0.1
Hypertension, *n* (%)	16 (30)	42 (55)	**0** **.** **006**
Diatetes, *n* (%)	10 (19)	23 (30)	0.2
Thyroid disease, *n* (%)	2 (4)	3 (4)	0.7
COVID during pregnancy, *n* (%)		3 (4)	
**Perinatal events**			
Chorioamnionitis, *n* (%)	14 (26)	16 (21)	0.5
Abnormal Fetal Heart rate tracing, *n* (%)	18 (33)	27 (35)	0.8
Acute abruption, *n* (%)	3 (6)	2 (3)	0.3
Uterine Rupture, *n* (%)	1 (2)	0	0.4
Cord prolapse, *n* (%)	2 (4)	0	0.2
Shoulder dystocia, *n* (%)	3 (6)	2 (3)	0.3
Urgent/Emergency C/S, *n* (%)	26 (48)	30 (39)	0.3
**Infant Demographics**			
GA, week, Median (IQR)	39.1 (38, 40.4)	39.0 (37.7, 39.6)	0.08
Birth weight, gram, Mean (SD)	3,485 (631)	3,188 (548)	**0** **.** **005**
Male, *n* (%)	28 (52)	41 (53)	0.9
Small for gestational age <10%ile, *n* (%)	2 (4)	9 (12)	0.09
Large for gestational age >90%ile, *n* (%)	9 (17)	6 (8)	0.1
**Delivery room outcomes**			
DR Intubation, *n* (%)	4 (7)	3 (4)	0.3
DR Chest compressions, *n* (%)	6 (11)	1 (1)	**0** **.** **02**
1 min APGAR, Median (IQR)	3.5 (2, 6)	5 (2, 7)	**0** **.** **02**
5 min APGAR, Median (IQR)	6.5 (5, 8)	8 (6, 9)	**0** **.** **002**
5 min APGAR ≤5, *n* (%)	16 (30)	16 (21)	**0** **.** **2**
**Critical cord or infant first hour blood gas**			
pH <7, *n* (%)	20 (37)	36 (47)	0.3
BD ≥ 16 mmol/L, *n* (%)	14 (26)	41 (53)	**0** **.** **002**
BD ≥ 12 mmol/L, *n* (%)	22 (41)	55 (71)	**<0** **.** **0001**
Abnormal neurological examination >1 h of life, *n* (%)	70	58	0.9
Multiorgan injury, *n* (%)	13 (24)	15 (20)	0.5
Early onset of sepsis, *n* (%)	0	0	

GA, gestational age; DR, delivery room; BD, base deficit.

The bold values are statistically significant (*p* < 0.05).

**Table 3:** Characteristics and outcomes of infants treated with therapeutic hypothermia.

	Pre-COVID-19 (2018–2019) *N* = 12	COVID-19 (2020–2021) *N* = 18	*p*-value
**HIE**			
Mild, *n* (%)	4 (33)	4 (22)	0.7
Moderate and Severe, *n* (%)	8 (67)	14 (78)	
Seizures, *n* (%)	1 (8)	4 (22)	0.3
**Brain MRI severity score, *n* (%)**			
0	7 (58)	15 (83)	0.2
1	3 (25)	1 (6)	
2	1 (8)	0	
3	1 (8)	2 (11)	
Multiorgan injury, *n* (%)	8 (67)	10 (56)	0.5
Death (NICU), *n* (%)	1 (8)	0	0.4
LOS, day, Median (IQR)	9 (7.5, 19.5)	10.5 (7, 16)	0.8
**At discharge, *n* (%)**			
Abnormal Neuro exam	0	3 (17)	0.2
Anti-seizure medication	0	0	
G-tube	0	0	

HIE, hypoxic ischemic encephalopathy; LOS, length of stay.

## Discussion

In this study, we examine the effect of COVID-19 on neonatal HIE and TH. During the COVID-19 pandemic, we observed a significant increase in maternal hypertension and infants with severe cord blood metabolic acidosis and abnormal neurological status at birth, resulting in a 40% increase in NICU admission for HIE evaluation. We did not find a significant difference in infants diagnosed with HIE or treated with TH between the two periods.

Universal screening of antenatal and perinatal risk factors for hypoxic-ischemic injury and recognition of the signs and symptoms of neonatal encephalopathy is essential for early identification and initiation of TH for all eligible newborns (12, 37). Our institution developed and implemented a neonatal HIE screening protocol based on the published criteria for TH (6) in 2008. Between 2008 and 2020, our annual rates of infants admitted to NICU for evaluation of HIE were within the range of 0.6%–1.1%. The rates started to trend above the mean level in 2019. However, they did not become statistically significant until 2021, when the rate reached 1.6%. During 2019–2021, there were no practice changes in obstetric delivery management, pediatric DR resuscitation, or HIE screening. This change is likely related to the COVID-19 pandemic, which started in early 2020 in the US. The pandemic has had an unprecedented negative impact on public health and well-being. The adverse effect was more profound in racial and ethnic minority groups and people with lower socio-economic status. As a public safety-net hospital, over 70% of our pregnant mothers are Hispanic, and a majority of them with significant socio-economic disparities. The up trend in infants requiring HIE evaluation indicates a progressive increase in infants with severe metabolic acidosis and abnormal neurological status at birth. In 2021, the second year of the COVID-19 pandemic, the rate of HIE evaluation was significantly above the historical level, suggesting that women who experienced the negative impact of the pandemic during their entire pregnancy had worse birth outcomes.

Maternal hypertension may be a significant contributing factor to the increased rate of NICU admission for HIE evaluation during the pandemic. Maternal hypertension, if not well controlled, causes poor placental perfusion and fetal growth, which in turn increases the risk for fetal intolerance during labor, sentinel events, metabolic acidosis, birth asphyxia, hypoxic-ischemic injury and neonatal HIE (27–30, 38–40). According to the 2022 CDC report, the overall prevalence of maternal hypertension in the US was 14.6% during 2017–2019, and the prevalence in Hispanic women was 12.5% (41). Our institution's pre-COVID-19 (2018–2019) maternal hypertension rate was 22% and increased to 26% during the pandemic. Notably, the maternal hypertension rate for those requiring HIE evaluation increased from 30% in the pre-pandemic to 55% during the pandemic. The pandemic could have exacerbated maternal hypertension and negatively impacted newborns' metabolic and neurological status. There are multiple reasons for maternal hypertension increase during the pandemic. Our patient population experienced significant financial, physical, and mental stress. Their access to routine, in-person health services and prenatal care were interrupted. They had limited resources for adapting to changes in the health care system, including online health services. All these could have contributed to less monitoring and treatment of chronic diseases and pregnancy-related complications. Similar to our study, Rao et al. (19) conducted a retrospective cohort study in a tertiary medical center in New York City, an epicenter of the pandemic. They found that women who delivered during the pandemic (27 March–31 May 2020) had a significantly higher rate of hypertensive disorders of pregnancy in maternal hypertension compared to women who delivered prior to the pandemic (27 March–31 May 2019) (OR = 1.05–1.85). Molina et al. (42) analyzed data from more than 1.6 million pregnant patients who gave birth in 463 US hospitals before and during the pandemic. They found a small but statistically significant increase in hypertensive disorders of pregnancy during the pandemic (OR, 1.04–1.08). A meta-analysis including 40 publications in 2020–2021 did not show a change in maternal hypertension during the pandemic (14). The discrepancy across the studies may reflect the difference in the timing and duration of studies, social environment due to infection control measures, and study populations.

Among the infants admitted to NICU for HIE evaluation, the average BW was significantly lower in the COVID-19 than in the pre-COVID-19 pandemic period (3,485 grams vs. 3,199 grams). The percentage of SGA infants increased from 2% in the pre-pandemic to 9% during the pandemic but was not statistically significant. BW is mainly determined by the duration of gestation and intrauterine growth. In this study, we included infants born at ≥35 weeks gestation, and the average GA of the infants admitted to NICU was not different between the two periods. Poor uteroplacental blood perfusion is the common pathophysiologic mechanism of intrauterine growth restriction (43–47). Furthermore, a fetus with placental insufficiency already suffers a baseline oxygen deficit at rest and has poor tolerance for labor. The superimposed hypoxic stress by uterine contractions during delivery can further worsen hypoxia and acidosis. In addition to maternal hypertension, other risk factors, such as, poor nutrition, prenatal maternal psychological distress (depression, anxiety, and stress) and substance use disorder, could have contributed to the low BW and SGA during the pandemic.

In our study, the pre-pandemic group had a higher rate of chest compressions. However, it only reflected six infants; three had brief chest compression and did not require intubation. The pre-pandemic group had less number of infants with severe metabolic acidosis but had overall low Apgar scores, even though the percentage of Apgar scores ≤5 was comparable to the pandemic period. Apgar scores and CBG are both commonly used in the newborn assessment (48, 49). CBG and acid-base balance, the most objective determinations of fetal hypoxia and metabolic condition at birth, are essential for diagnosing asphyxia and HIE (6, 48). The Apgar score provides an accepted and convenient method for reporting the status of the newborn infant immediately after birth and the response to resuscitation. While low Apgar scores may be one of the first indications of neonatal encephalopathy, its alone cannot be considered as the evidence or consequence of asphyxia. It does not predict individual neonatal mortality or neurologic outcome. In population-based studies, 5- and 10-minute Apgar scores ≥ five confer a clear increased risk of cerebral palsy (49). Several studies have shown a poor correlation between 1- and 5-minute Apgar scores and neonatal acid-base status (50–53). In addition to asphyxia, low Apagar scores can result from genetic diseases, congenital anomalies, maternal mediation during delivery, acute airway obstruction, as well as many other prenatal, perinatal, and postnatal factors that may not present with metabolic acidosis at birth. On the other hand, some neonates with reassuring Apgar scores still have a risk of CBG acidemia and poor birth outcomes (52, 54).

The global incidence of neonatal encephalopathy varies between 1 and 8 per 1,000 live births (1). Moderate/severe neonatal encephalopathy affects 0.5–3/1,000 live births in high-income countries, but higher in low- and middle-income countries (1, 55). In our patient population, the rate of HIE requiring TH has been relatively stable at 1–4/1,000 live births over the past 14 years. During the pandemic, the numbers of infants for HIE evaluation increased by 40% but the number of infants who met the diagnosed of HIE did not change significantly. This is because some infants who met the HIE screening criteria based on their cord blood acidosis had transient abnormal neurological examination, which was normalized within the first hour of life, hence were not diagnosed with HIE. While the severity and outcomes of the infants who were treated with TH were comparable between the two periods, the number of cases are very small. Few studies have assessed the impact of the COVID-19 pandemic on neonatal HIE. A single-center study found that more infants were diagnosed with HIE and treated with hypothermia during the first wave of the pandemic in Turkey (56). Similarly, a higher incidence of HIE was observed in a large NICU in the UK during the pandemic (57). Data from level-3 NICUs in the Canadian Neonatal Network (58). showed increased HIE and TH treatment during pandemic lockdown. However, the severity of HIE, associated morbidities, and mortality were not significantly different during the pandemic. Since this study used the hospital data from level-3 cooling centers, it is unclear whether the overall incidence of HIE, including mild HIE and their outcomes were affected by the pandemic.

TH is a standard of care therapy for infants with moderate and severe HIE. However, the risk-benefit balance of TH in mild HIE remains to be determined (37). In our study, mild HIE accounted for 33% and 22% of the TH cases in the pre-pandemic and pandemic periods, respectively. The decision to cool mild HIE cases was based on our evaluation protocol, which did not change during the study periods. In our practice, infants at risk for HIE but who do not meet the criteria for TH immediately after birth continue to be evaluated during the first five hours of life. TH treatment is used in infants with persistent mild abnormal or worsening neurological examinations, abnormal aEEG (with raw EEG tracing), or laboratory tests showing evidence of multiorgan injuries.

Our study has several limitations. It is a single-center retrospective study. While we observed a significant increase in NICU admission for HIE evaluation during the pandemic, other factors might have contributed to this change. It is important to continue monitoring the trend and identify other possible underlying causes for improving maternal and infant outcomes. Our sample sizes of HIE evaluation and TH are small. More studies with larger sample sizes are needed to confirm our findings.

## Conclusion

During the COVID-19 pandemic, we observed a significant increase infants with severe metabolic acidosis and abnormal neurologic status at birth, resulting in an increased NICU admission for HIE evaluation. Increased maternal hypertension during the pandemic may be a significant contributing factor. The adverse effect of the COVID-19 pandemic on maternal morbidities may persist beyond the pandemic and should be closely monitored. While this single canter data did not show significant increases in neonatal HIE and the need for therapeutic hypothermia, larger studies and meta-analyses are needed for a comprehensive assessment of the impact of the COVID-19 pandemic on neonatal HIE.

## Data Availability

The raw data supporting the conclusions of this article will be made available by the authors, without undue reservation.

## References

[1] Lee AC Kozuki N Blencowe H Vos T Bahalim A Darmstadt GL Intrapartum-related neonatal encephalopathy incidence and impairment at regional and global levels for 2010 with trends from 1990. Pediatr Res. (2013) 74(Suppl 1):50–72. 10.1038/pr.2013.206 24366463 PMC3873711

[2] Perin J Mulick A Yeung D Villavicencio F Lopez G Strong KL Global, regional, and national causes of under-5 mortality in 2000–19: an updated systematic analysis with implications for the sustainable development goals. Lancet Child Adolesc Health. (2022) 6(2):106–15. 10.1016/S2352-4642(21)00311-4 34800370 PMC8786667

[3] Schreglmann M Ground A Vollmer B Johnson MJ. Systematic review: long-term cognitive and behavioural outcomes of neonatal hypoxic-ischaemic encephalopathy in children without cerebral palsy. Acta Paediatr. (2020) 109(1):20–30. 10.1111/apa.14821 31002422

[4] Marlow N Shankaran S Rogers EE Maitre NL Smyser CD, Committee NBSGaP. Neurological and developmental outcomes following neonatal encephalopathy treated with therapeutic hypothermia. Semin Fetal Neonatal Med. (2021) 26(5):101274. 10.1016/j.siny.2021.101274 34330680

[5] Executive summary: Neonatal encephalopathy and neurologic outcome, second edition. Report of the American college of obstetricians and Gynecologists’ task force on neonatal encephalopathy. Obstet Gynecol. (2014) 123(4):896–901. 10.1097/01.AOG.0000445580.65983.d2 24785633

[6] Shankaran S Laptook AR Ehrenkranz RA Tyson JE McDonald SA Donovan EF Whole-body hypothermia for neonates with hypoxic-ischemic encephalopathy. N Engl J Med. (2005) 353(15):1574–84. 10.1056/NEJMcps050929 16221780

[7] Gluckman PD Wyatt JS Azzopardi D Ballard R Edwards AD Ferriero DM Selective head cooling with mild systemic hypothermia after neonatal encephalopathy: multicentre randomised trial. Lancet. (2005) 365(9460):663–70. 10.1016/S0140-6736(05)17946-X 15721471

[8] Zhou WH Cheng GQ Shao XM Liu XZ Shan RB Zhuang DY Selective head cooling with mild systemic hypothermia after neonatal hypoxic-ischemic encephalopathy: a multicenter randomized controlled trial in China. J Pediatr. (2010) 157(3):367–72. 72.e1-3. 10.1016/j.jpeds.2010.03.030 20488453

[9] Jacobs SE Berg M Hunt R Tarnow-Mordi WO Inder TE Davis PG. Cooling for newborns with hypoxic ischaemic encephalopathy. Cochrane Database Syst Rev. (2013) 1:CD003311. 10.1002/14651858.CD003311.pub3 PMC7003568 23440789

[10] Wassink G Davidson JO Dhillon SK Zhou K Bennet L Thoresen M Therapeutic hypothermia in neonatal hypoxic-ischemic encephalopathy. Curr Neurol Neurosci Rep. (2019) 19(2):2. 10.1007/s11910-019-0916-0 30637551

[11] Thoresen M Tooley J Liu X Jary S Fleming P Luyt K Time is brain: starting therapeutic hypothermia within three hours after birth improves motor outcome in asphyxiated newborns. Neonatology. (2013) 104(3):228–33. 10.1159/000353948 24030160

[12] Jegatheesan P Morgan A Shimotake T Song D Van Meurs K. Early screening and identification of candidates for neonatal therapeutic hypothermia toolkit. In: (PQIP) PQIP, (CPQCC) CPQCC, editors. California Perinatal Quality Care Collaborative2015.

[13] Villar J Ariff S Gunier RB Thiruvengadam R Rauch S Kholin A Maternal and neonatal morbidity and mortality among pregnant women with and without COVID-19 infection: the INTERCOVID multinational cohort study. JAMA Pediatr. (2021) 175(8):817–26. 10.1001/jamapediatrics.2021.1050 33885740 PMC8063132

[14] Chmielewska B Barratt I Townsend R Kalafat E van der Meulen J Gurol-Urganci I Effects of the COVID-19 pandemic on maternal and perinatal outcomes: a systematic review and meta-analysis. Lancet Glob Health. (2021) 9(6):e759–72. 10.1016/S2214-109X(21)00079-6 33811827 PMC8012052

[15] Wang H Li N Sun C Guo X Su W Song Q The association between pregnancy and COVID-19: a systematic review and meta-analysis. Am J Emerg Med. (2022) 56:188–95. 10.1016/j.ajem.2022.03.060 35413655 PMC8986277

[16] Ryan L Plötz FB van den Hoogen A Latour JM Degtyareva M Keuning M Neonates and COVID-19: state of the art: neonatal sepsis series. Pediatr Res. (2022) 91(2):432–9. 10.1038/s41390-021-01875-y 34961785 PMC8712275

[17] Kotlar B Gerson E Petrillo S Langer A Tiemeier H. The impact of the COVID-19 pandemic on maternal and perinatal health: a scoping review. Reprod Health. (2021) 18(1):10. 10.1186/s12978-021-01070-6 33461593 PMC7812564

[18] Masters GA Asipenko E Bergman AL Person SD Brenckle L Moore Simas TA Impact of the COVID-19 pandemic on mental health, access to care, and health disparities in the perinatal period. J Psychiatr Res. (2021) 137:126–30. 10.1016/j.jpsychires.2021.02.056 33677216 PMC8084993

[19] Rao MG Toner LE Stone J Iwelumo CA Goldberger C Roser BJ Pregnancy during a pandemic: a cohort study comparing adverse outcomes during and before the COVID-19 pandemic. Am J Perinatol. (2023) 40(4):445–52. 10.1055/a-1877-5973 35709734

[20] Auger N Wei SQ Dayan N Ukah UV Quach C Lewin A Impact of COVID-19 on rates of gestational diabetes in a North American pandemic epicenter. Acta Diabetol. (2022) 60(2):1–8. 10.1007/s00592-022-02000-z 36346488 PMC9640820

[21] DeBolt CA Roig J Spiera E Goldberger C Kaplowitz E Toner L The impact of the COVID-19 pandemic on postpartum readmission rates at a single tertiary care center in New York city. Am J Perinatol. (2022) 39(11):1145–50. 10.1055/a-1774-5969 35176782

[22] Zheng W Wang J Zhang K Liu C Zhang L Liang X Maternal and infant outcomes in women with and without gestational diabetes mellitus in the COVID-19 era in China: lessons learned. Front Endocrinol. (2022) 13:982493. 10.3389/fendo.2022.982493 PMC9723325 36482992

[23] Zanardo V Tortora D Guerrini P Garani G Severino L Soldera G Infant feeding initiation practices in the context of COVID-19 lockdown. Early Hum Dev. (2021) 152:105286. 10.1016/j.earlhumdev.2020.105286 33276222 PMC7690304

[24] Ornaghi S Fumagalli S Guinea Montalvo CK Beretta G Invernizzi F Nespoli A Indirect impact of SARS-CoV-2 pandemic on pregnancy and childbirth outcomes: a nine-month long experience from a university center in lombardy. Int J Gynaecol Obstet. (2022) 156(3):466–74. 10.1002/ijgo.13990 34669973 PMC9087530

[25] Mirsky EL Mastronardi AM Paudel A Young ML Zite NB Maples JM. The COVID-19 pandemic and prevalence of gestational diabetes: does gestational weight gain matter? Am J Obstet Gynecol MFM. (2023) 5(5):100899. 10.1016/j.ajogmf.2023.100899 36764456 PMC9908741

[26] Wei SQ Bilodeau-Bertrand M Liu S Auger N. The impact of COVID-19 on pregnancy outcomes: a systematic review and meta-analysis. CMAJ. (2021) 193(16):E540–E8. 10.1503/cmaj.202604 33741725 PMC8084555

[27] Badawi N Kurinczuk JJ Keogh JM Alessandri LM O’Sullivan F Burton PR Intrapartum risk factors for newborn encephalopathy: the Western Australian case-control study. Br Med J. (1998) 317(7172):1554–8. 10.1136/bmj.317.7172.1554 9836653 PMC28733

[28] Badawi N Kurinczuk JJ Keogh JM Alessandri LM O’Sullivan F Burton PR Antepartum risk factors for newborn encephalopathy: the Western Australian case-control study. Br Med J. (1998) 317(7172):1549–53. 10.1136/bmj.317.7172.1549 9836652 PMC28732

[29] Aslam S Strickland T Molloy EJ. Neonatal encephalopathy: need for recognition of multiple etiologies for optimal management. Front Pediatr. (2019) 7:142. 10.3389/fped.2019.00142 31058120 PMC6477286

[30] Yang W Wang L Tian T Liu L Jin L Liu J Maternal hypertensive disorders in pregnancy and risk of hypoxic-ischemia encephalopathy. J Matern Fetal Neonatal Med. (2021) 34(11):1754–62. 10.1080/14767058.2019.1647529 31331218

[31] O’Dea M Sweetman D Bonifacio SL El-Dib M Austin T Molloy EJ. Management of multi organ dysfunction in neonatal encephalopathy. Front Pediatr. (2020) 8:239. 10.3389/fped.2020.00239 32500050 PMC7243796

[32] Barkovich AJ Hajnal BL Vigneron D Sola A Partridge JC Allen F Prediction of neuromotor outcome in perinatal asphyxia: evaluation of MR scoring systems. AJNR Am J Neuroradiol. (1998) 19(1):143–9. 9432172 PMC8337350

[33] Gestational hypertension and preeclampsia: aCOG practice bulletin, number 222. Obstet Gynecol. (2020) 135(6):e237–60. 10.1097/AOG.0000000000003891 32443079

[34] Bulletins—Obstetrics ACoOaGCoP. ACOG practice bulletin No. 203: chronic hypertension in pregnancy. Obstet Gynecol. (2019) 133(1):e26–50. 10.1097/01.AOG.0000559202.88943.30 30575676

[35] ACOG practice bulletin No. 190: gestational diabetes mellitus. Obstet Gynecol. (2018) 131(2):e49–64. 10.1097/AOG.0000000000002501 29370047

[36] Bulletins—Obstetrics ACoOaGCoP. ACOG practice bulletin No. 201: pregestational diabetes Mellitus. Obstet Gynecol. (2018) 132(6):e228–48. 10.1097/AOG.0000000000002960 30461693

[37] Bonifacio SL Hutson S. The term newborn: evaluation for hypoxic-ischemic encephalopathy. Clin Perinatol. (2021) 48(3):681–95. 10.1016/j.clp.2021.05.014 34353587

[38] Nelson KB Bingham P Edwards EM Horbar JD Kenny MJ Inder T Antecedents of neonatal encephalopathy in the Vermont Oxford network encephalopathy registry. Pediatrics. (2012) 130(5):878–86. 10.1542/peds.2012-0714 23071210 PMC4074646

[39] Parker SJ Kuzniewicz M Niki H Wu YW. Antenatal and intrapartum risk factors for hypoxic-ischemic encephalopathy in a US birth cohort. J Pediatr. (2018) 203:163–9. 10.1016/j.jpeds.2018.08.028 30270166

[40] Bandoli G Suttner D Kiernan E Baer RJ Jelliffe-Pawlowski L Chambers CD. Risk factors for neonatal encephalopathy in late preterm and term singleton births in a large California birth cohort. J Perinatol. (2022) 42(3):341–7. 10.1038/s41372-021-01242-z 34702969 PMC8917979

[41] Ford ND Cox S Ko JY Ouyang L Romero L Colarusso T Hypertensive disorders in pregnancy and mortality at delivery hospitalization - United States, 2017–2019. MMWR Morb Mortal Wkly Rep. (2022) 71(17):585–91. 10.15585/mmwr.mm7117a1 35482575 PMC9098235

[42] Molina RL Tsai TC Dai D Soto M Rosenthal N Orav EJ Comparison of pregnancy and birth outcomes before vs during the COVID-19 pandemic. JAMA Netw Open. (2022) 5(8):e2226531. 10.1001/jamanetworkopen.2022.26531 35960517 PMC9375166

[43] Macdonald-Wallis C Tilling K Fraser A Nelson SM Lawlor DA. Associations of blood pressure change in pregnancy with fetal growth and gestational age at delivery: findings from a prospective cohort. Hypertension. (2014) 64(1):36–44. 10.1161/HYPERTENSIONAHA.113.02766 24821945 PMC4150069

[44] Madden JV Flatley CJ Kumar S. Term small-for-gestational-age infants from low-risk women are at significantly greater risk of adverse neonatal outcomes. Am J Obstet Gynecol. (2018) 218(5):525.e1–.e9. 10.1016/j.ajog.2018.02.008 29462628

[45] Bligh LN Flatley CJ Kumar S. Reduced growth velocity at term is associated with adverse neonatal outcomes in non-small for gestational age infants. Eur J Obstet Gynecol Reprod Biol. (2019) 240:125–9. 10.1016/j.ejogrb.2019.06.026 31265938

[46] Liu Y Li N An H Li Z Zhang L Li H Impact of gestational hypertension and preeclampsia on low birthweight and small-for-gestational-age infants in China: a large prospective cohort study. J Clin Hypertens (Greenwich). (2021) 23(4):835–42. 10.1111/jch.14176 33507600 PMC8678768

[47] Avorgbedor F Silva S McCoy TP Blumenthal JA Merwin E Seonae Y Hypertension and infant outcomes: North Carolina pregnancy risks assessment monitoring system data. Pregnancy Hypertens. (2022) 28:189–93. 10.1016/j.preghy.2022.05.004 35576746

[48] Practice ACoO. ACOG committee opinion No. 348, November 2006: umbilical cord blood gas and acid-base analysis. Obstet Gynecol. (2006) 108(5):1319–22. 10.1097/00006250-200611000-00058 17077266

[49] Committee opinion No. 644: the Apgar score. Obstet Gynecol. (2015) 126(4):e52–5. 10.1097/AOG.0000000000001108 26393460

[50] Sykes GS Molloy PM Johnson P Gu W Ashworth F Stirrat GM Do Apgar scores indicate asphyxia? Lancet. (1982) 1(8270):494–6. 10.1016/S0140-6736(82)91462-3 6121150

[51] Silverman F Suidan J Wasserman J Antoine C Young BK. The Apgar score: is it enough? Obstet Gynecol. (1985) 66(3):331–6. 3927209

[52] Sabol BA Caughey AB. Acidemia in neonates with a 5-minute Apgar score of 7 or greater - what are the outcomes? Am J Obstet Gynecol. (2016) 215(4):486.e1–6. 10.1016/j.ajog.2016.05.035 27255470

[53] Njie AE Nyandiko WM Ahoya PA Moutchia JS. A comparative analysis of APGAR score and the gold standard in the diagnosis of birth asphyxia at a tertiary health facility in Kenya. PLoS One. (2023) 18(5):e0285828. 10.1371/journal.pone.0285828 37224111 PMC10208496

[54] Malin GL Morris RK Khan KS. Strength of association between umbilical cord pH and perinatal and long term outcomes: systematic review and meta-analysis. Br Med J. (2010) 340:c1471. 10.1136/bmj.c1471 20466789 PMC2869402

[55] Kurinczuk JJ White-Koning M Badawi N. Epidemiology of neonatal encephalopathy and hypoxic-ischaemic encephalopathy. Early Hum Dev. (2010) 86(6):329–38. 10.1016/j.earlhumdev.2010.05.010 20554402

[56] Hekimoğlu B Aktürk Acar F. Effects of COVID-19 pandemic period on neonatal mortality and morbidity. Pediatr Neonatol. (2022) 63(1):78–83. 10.1016/j.pedneo.2021.08.019 34776364 PMC8548836

[57] Siddhi PS Kollurage U Fassaludhin N Price MJ. Impact of COVID-19 on neonatal outcomes. Pediatr Neonatol. (2022) 63(4):436. 10.1016/j.pedneo.2022.03.005 35437229 PMC8971058

[58] Gurram Venkata SKR Shah PS Beltempo M Yoon E Wood S Hicks M Outcomes of infants with hypoxic-ischemic encephalopathy during COVID-19 pandemic lockdown in Canada: a cohort study. Childs Nerv Syst. (2022) 38(9):1727–34. 10.1007/s00381-022-05575-8 35676388 PMC9177131

